# Improving child health service interventions through a Theory of Change: A scoping review

**DOI:** 10.3389/fped.2023.1037890

**Published:** 2023-04-06

**Authors:** Benjamin Jones, Amy Paterson, Mike English, Shobhana Nagraj

**Affiliations:** ^1^ Health Systems Collaborative, Centre for Global Health Research, Nuffield Department of Medicine, University of Oxford, United Kingdom; ^2^ Oxford University Global Surgery Group, Nuffield Department of Surgical Sciences, University of Oxford, Oxford, United Kingdom; ^3^ KEMRI-Wellcome Trust Research Programme, Kilifi, Kenya

**Keywords:** health service intervention, quality improvement, implementation sceince, evaluation, program theory, program design, theory of change

## Abstract

**Background:**

The objective of this scoping review was to map how child health service interventions develop, utilise, and refine theories of change. A Theory of Change (ToC) is a tool for designing, implementing, and evaluating interventions that is being increasingly used by child health practitioners who are aiming to enact change in health services.

**Methods:**

A published protocol guided this scoping review. Relevant publications were identified through selected electronic databases and grey literature *via* a search strategy. The main inclusion criteria were any child health service intervention globally that described their ToC or ToC development process. These were applied by two independent reviewers. Data relevant to the research sub-questions were extracted, charted and discussed.

**Findings:**

38 studies were included in the analysis. This scoping review highlights the disparate and inconsistent use, and reporting of ToCs in the child health service intervention literature.

**Conclusion:**

A ToC may be a helpful tool to enact change in a child health service but careful consideration must be undertaken by the child health service regarding how to maximise the benefits of doing a ToC, and how to accurately report it.

## 1. Introduction

Child health service interventions (CHSI) target the access to, and the use, costs, quality, delivery, organisation, financing, and outcomes of child healthcare services (1–3). These interventions can be any organised activity, program, project, or initiative, that is supported by resources and established with the purpose of enacting change for children aged 0–19 years old. They can be based in the community, primary-care or hospital, with a child health focus, and may encompass multiple components including, healthcare access, human resourcing, training and education, health facility processes and policies, quality improvement, e-health, task shifting, and healthcare continuity.

CHSI often involve multiple stakeholders (e.g., parents and schools), diverse objectives (e.g., health and education), and are dependent on context, making them inherently complex. As this complexity is increasingly being recognised by stakeholders in child health including practitioners, researchers, and funders, there is more of a need to articulate the ways in which this complexity has been considered in the design, implementation, and evaluation of CHSI. A Theory of Change (ToC) is a tool that child health practitioners leading such interventions can use to navigate and report on this complexity. For example, child health practitioners developed a ToC for their 2020 BetterBirth Program which included leadership engagement, an educational and motivational program launch, and ongoing coaching visits all aiming to promote the use of the World Health Organization's Safe Childbirth Checklist (4). Similarly, the child health practitioners for The Future Health Systems Program, published their ToC in 2017 to demonstrate how they navigated the complexity of improving information systems and coordination of child health service providers in India (5).

A ToC is a an articulation of the hypothesised pathways of how and why an intervention is intended to bring about change (6–9). It presents an intervention's assumptions, activities, mechanisms, measurement indicators, outcomes, and context, as well as the linkages between these various components (7). For child health practitioners looking to develop or evaluate an intervention, a ToC can be used as a guiding tool for reflecting and making explicit these important components of an intervention. Doing so allows teams to better understand if an intervention is working in the way that they had hypothesised, and assess implementation successes and failures. It also allows for better communication of the intervention to community, colleagues and others looking to carry out, or scale up, similar interventions, and to stakeholders in order to obtain more resources. These benefits have been recognised by the Medical Research Council, UK, who highlight the value of developing program theory in their 2021 framework for developing and evaluating complex interventions (10).

Whilst ToC is one of many tools used to better understand an intervention, it differs from other theories, and frameworks and models in several ways. Firstly, in its focus, a ToC provides an *explanation* of how and why a specific intervention leads to change (6, 7, 9). This differentiates it from frameworks (e.g., RE-AIM) and models (e.g., PDSA) as frameworks and models do not explore explanations but focus on *describing* intervention implementation aspects or endeavours (often without exploring the relationship between these aspects) and by structuring them into categories (11). Secondly, ToC has a particular focus on causality and outlines the steps in the causal chain or logic that connects an intervention's activities to its outcomes (6, 7, 9). As per De Silva, Breuer, Lee et al. (9), theories that exist at a more abstract level (e.g., Normalisation Process Theory) can strengthen ToCs by contributing a theoretical basis of why particular casual links happen. Finally, a ToC differs in that it can be utilised at any stage of an intervention from planning to evaluating (6, 7, 9).

The origins of ToC, and the overarching field, theory-based evaluation were popularised in the 1990s (7, 8, 12–14). They have been used in many contexts, including health, education, business, social welfare and international development (6, 9, 13, 14). Benefits of the ToC development process include, engaging stakeholders, clarifying focus, and enhancing connection to the intervention. Benefits once completed include, providing a roadmap for the intervention, a clear anchor for evaluation, a base in which to refine thinking and monitor the intervention as context changes overtime, scale-up utility, and identifying intentional and unintentional consequences (7, 9, 13–17). Criticisms of ToC include, confusion around definition, the so-called reductionist modelling which may give a false sense of control to the intervention implementers, a lack of enforced academic rigour, and the provision of an excuse not to adapt when context changes (7–9, 13).

This scoping review aims to introduce readers to ToC and explore how they have been developed, used, and refined in the CHSI context, and discuss areas where child health organisations and practitioners may improve their use of this tool and improve health service interventions more broadly. Specifically, this scoping review aims to answer the following question—how have ToCs for CHSI been developed, utilised, and refined? It will also aim to answer the following research sub-questions:

• How do these studies define ToC?
• What is the rationale for the ToC being developed?
• What is the process of development of the ToC?
• Who is involved in the development of the ToC?
• At what stage in the intervention are ToCs developed?
• How are the ToCs presented in the literature?
• In what way is the ToC used (purpose)?
• Is the value of the ToC outlined, and if so what is it and the evidence supporting it?
• How is the ToC refined overtime?

## 2. Objectives

To map how CHSI ToCs have been developed, utilised, and refined.

## 3. Methods

A scoping review was selected for this study as it provides an opportunity to map an overview of the available research evidence. A protocol for this scoping review was registered (OSF, DOI 10.17605/OSF.IO/5TPGM), published and peer-reviewed in January, 2022 (18). This protocol was used as the methodological basis for this scoping review except where explicitly stated. This scoping review was conducted in accordance with the widely utilised JBI methodology (19, 20). A PRISMA-ScR checklist was also used to guide the study (Supplementary File S1).

## 4. Eligibility criteria

Table 1 outlines the review inclusion and exclusion criteria.

**Table 1:** Eligibility criteria for studies in this review.

Category	Inclusion criteria	Exclusion criteria
**Participants**	Studies were included if they: • Described child health service interventions^a^ targeted at children aged 0–19 years^b^	Studies were included if they: • Focused on obstetric health service interventions that aim to improve maternal health outcomes rather than newborn health outcomes. • Described interventions done exclusively in settings outside healthcare facilities such as schools. • Focused on interventions delivered by non-health related social services. • All other theory-based evaluation methods^c^ including logic models^d^ and realist evaluations were excluded.
**Concept**	Studies were included if they: • Described how a ToC, which was defined as a hypothesis of how and why an intervention is intended to bring about change, was utilised throughout any stage of a child health service intervention such as design, implementation, or evaluation. • Described the development process for a ToC planned to be used in a child health service intervention. • The ToC could be in narrative form or illustrated visually using ToC diagrams.	
**Context**	• Studies conducted anywhere globally were included. • There were no restrictions in terms of the date of the study.	
**Sources**	This scoping review included: • Quantitative, qualitative and mixed-methods study designs. • Organisational or other grey literature e.g., private organisations and NGO's as well as government ToC documents.	• Reviews of any kind
**Language**	• Included studies were restricted to the English language.	

^a^ Health service interventions were defined as interventions that are related to the health facility, or are delivered by healthcare providers directly. This includes public health interventions such as vaccination, nutrition, or preventative programs if they were delivered by health professionals. It does not include interventions done exclusively in settings outside healthcare facilities such as schools or delivered by other community non-health related social services.

^b^ If a study included an age range both within and outside the defined range the study was included if a majority (>50%) of the included years fall between 0 and 19 e.g. 10–22 would be included but 15–30 was not.

^c^ Due to confusion and common mislabelling, at the stage of title and abstract screening any form of theory-based evaluation, including logic model, program theory, outcomes hierarchy, theory of action, results chain, logic framework, or, studies that referred to the “how” and “why” of an intervention were included for full text review. The full-texts were then carefully screened using the specific definition of ToC, and only included in the analysis if they referred to several of the defining ToC components, namely assumptions, activities, mechanisms, measurement indicators, outcomes, and context, or the linkages between these various components.

^d^ Logic models, are, strictly speaking, more simple and linear than ToCs, that do not typically outline assumptions, measurement indicators or describe a consideration of the intervention's causal relationships (9). In this sense they are in some way a “partial” ToC but are often erroneously referred to as ToCs.

## 5. Search strategy

MEDLINE, EMBASE, Global Health, WHO Global Index Medicus, CINAHL and SCOPUS were searched from 1946—present. A search strategy was developed for each database (Supplementary File S2). The first 10 pages of the google search “Child* health* “theory of change” filetype:pdf” were searched for grey literature. The term “health*” was added and differs from the protocol. The database searches were conducted in January and February 2022 by BJ.

## 6. Evidence selection

**Figure 1:**
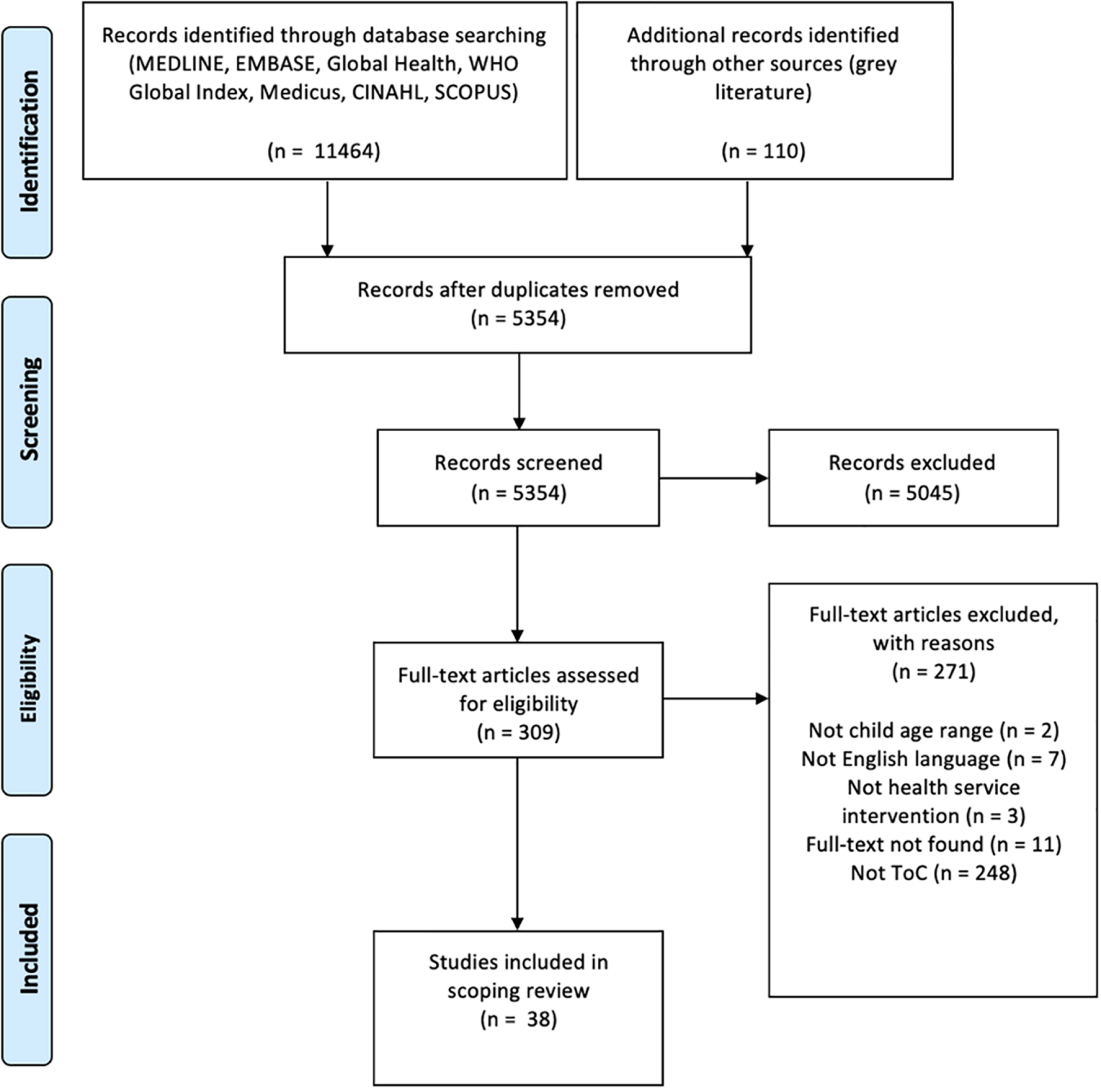
PRISMA-ScR flow diagram of search result.

Citations were uploaded to Endnote V20 and duplicates removed. Rayyan software (21) was used by two independent reviewers (BJ and AP) to screen titles and abstracts using the eligibility criteria. A screening guide was developed (Supplementary File S3) to aid in title and abstract screening. All conflicts between the reviewers were resolved without the need for a third reviewer. The guide was refined based on these conflicts for use in full text screening. All eligible full texts were reviewed by BJ. A random 20% of these were reviewed by an additional reviewer (AP). Any disagreements at each stage of the selection process were resolved through discussion or review by a third reviewer (SN). The results of the search and the study inclusion process are reported in the adapted Preferred Reporting Items for Systematic Reviews and Meta-analyses extension for scoping review (PRISMA-ScR) flow diagram (see Figure 1).

## 7. Data charting

Data were extracted from studies included in the scoping review by BJ using a data extraction tool developed and reviewed by all authors, to aid with consistency. The data extracted included: author, link to paper, year of publication, study country, name of intervention, ToC level, type of health service intervention, health service level, definition of ToC, process of development of ToC, stakeholders involved in ToC development, stage in which ToC was developed, ToC presentation in the literature, ToC purpose (articulation of why it is useful), ToC value (what it helped do), ToC refinement over time, and other. The components of the ToC were also extracted. In the absence of a gold standard ToC components checklist, a classification was been developed based on the work of Dhillon and Vaca (22) and Vogel (23). This classification of the components of a ToC, which uses the acronym *COMMA*, consists of the following elements; Context, Outcomes, Mechanisms, Measurement indicators, and Assumptions.

Raw data is presented in table form (Supplementary File S4). A narrative synthesis of the data was conducted using both deductive (pre-defined research questions) and inductive elements to look at consistent themes arising from the included studies. The literature was also quantitatively analysed for study location, type of health service intervention, ToC presentation, and ToC definition using a checklist of ToC components such as assumptions, activities, mechanisms, measurement indicators, outcomes, context, and linkages (see discussion for more detail of ToC components).

## 8. Results

### 8.1. Search results

5,354 abstracts and 309 full texts were screened using the eligibility criteria. A total of 38 full-texts were included for data extraction and analysis. This process is summarised in an adapted PRISMA-ScR model shown in Figure 1.

### 8.2. Included studies

**Figure 2:**
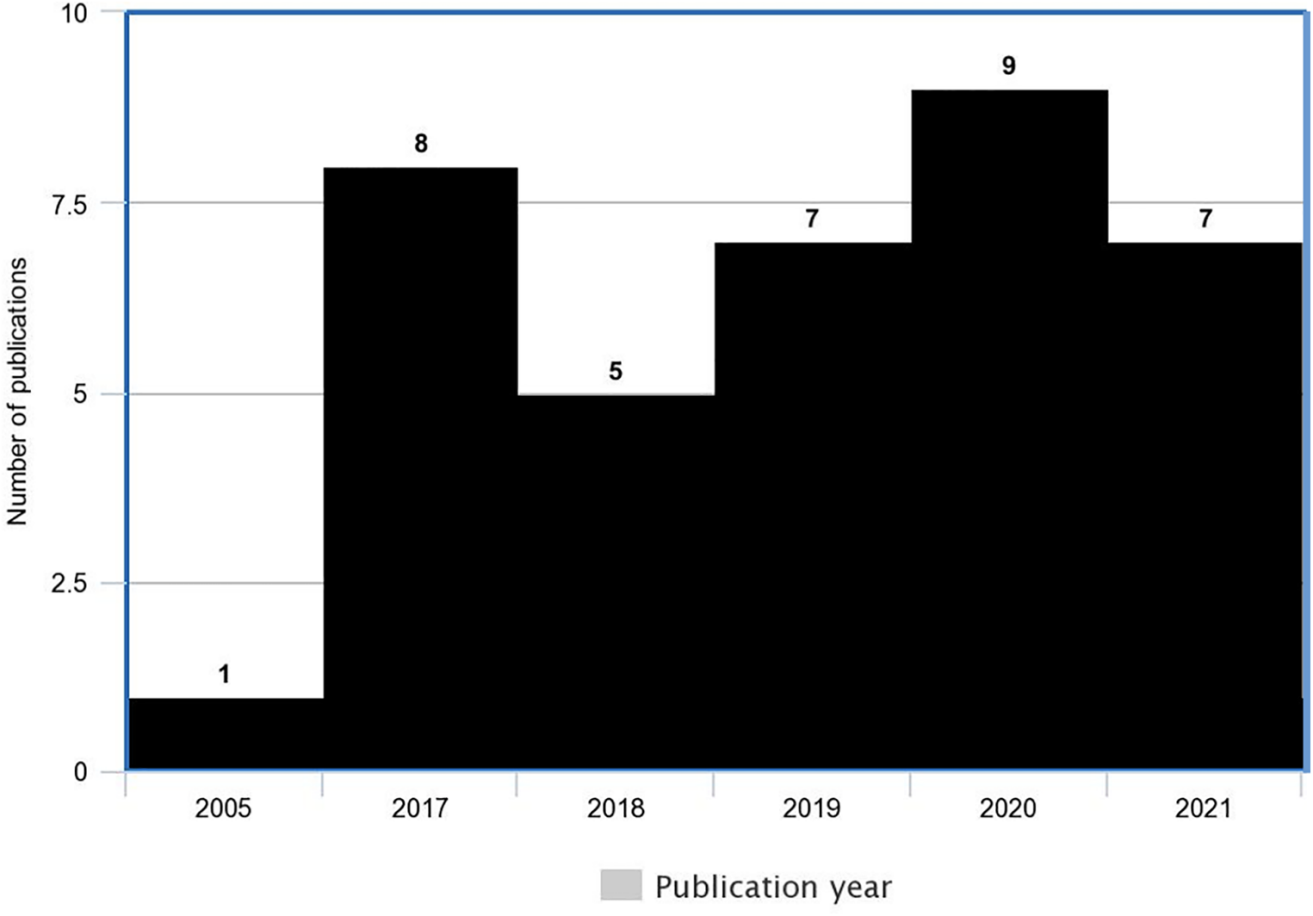
Histogram of number of publication per year (note 1 paper was also published in 20222).

The publication date of the included studies ranged from 2005 to 2022 with an increase in studies in the past five years (Figure 2). Of the 38 ToCs presented, 16 (42%) represented a single-component CHSI and 22 (58%) represented multi-component CHSIs and 15 (47%) included ToCs that encompassed multi-country sites. The majority of studies, [27/38 (71%)] were conducted in low- or middle-income countries, eight (21%) were in high-income countries, one across both and two did not specify where they were conducted. The most common countries were India (8 studies), Ethiopia (5 studies) and the UK (4 studies).

A number of different types of health service interventions were reported (Supplementary File S4). These include sexual and reproductive health, mental health, infectious disease, maternal and childhood nutrition, supply chain, home visitation, safe antenatal care and childbirth, violence prevention, teamwork related, weight management, pain treatment, and vaccination services. Of the 38 studies, 20 involved (53%) community-based interventions, seven (18%) only included hospital-based interventions, six (16%) only featured primary-care interventions, five (13%) involved interventions across multiple settings and one was a policy -based intervention.

### 8.3. ToC definition and presentation

Twenty four studies (63%) provided a definition for ToC or program theory. The most common elements in the definition were; “how” and/or “why” (24–31), “assumptions” (4, 24, 29, 32), “hypothesis” (4, 25, 26, 33, 34), and “tool” (4, 5, 35–37). 26 studies (68%) described the diagrammatic representation of their model as a “ToC diagram” with the remainder (32%) describing their diagrams as “logic models”, “program theory”, or “causal web diagrams”.

### 8.4. ToC development

Only 13 studies (34%) provided commentary on which stakeholders were involved in the ToC development process. Of these, two mentioned the community/beneficiaries of the intervention as stakeholders involved in the process (4, 38). Further, only 10 studies (26%) spoke to the refinement of ToCs over time—one of the most important aspects of a ToC (5, 29–31, 35, 37, 39–42).

Exactly half of the ToCs (19/38) appeared to be developed pre-intervention. Of the remainder, 9 (24%) were developed post-intervention, 8 (21%) during the intervention, and the timing of the development of two was not clear. Under half of the studies (16/38, 42%) commented on the process of ToC development, with many describing a collaborative approach through workshops, meetings, or discussions (5, 25, 30, 31, 33–35, 37, 38, 43, 44). Seven studies (18%) provided a stepwise and more comprehensive explanation for their ToC development (5, 25, 28, 33, 38, 39, 42).

### 8.5. ToC purpose and value

A justification for the ToC approach being used was identified in 14 studies (37%). Of these the most common reasons were monitoring and evaluation (4, 5, 28, 30, 31, 45), testing causal links (38, 40, 45, 46), and collaborating with stakeholders (4, 28, 35, 39). 16 studies (42%) included some form of reflection on the value of a ToC. This included comments on using data more effectively (4, 5, 47, 48), engaging stakeholders (5, 28, 33, 38, 48), and learning about the intervention (5, 29). However, there were some studies that reported more critical reflections, for example, that ToCs can be too linear and lack detail (26).

### 8.6. ToC components

ToC components and the frequency of use are reported in Table 2.

**Table 2:** Toc components.

ToC Component	Context	Outcomes	Mechanisms	Measurement indicators	Assumptions
Definition	The context of the intervention (including programme activities) and the context of the ToC process.	The pathway of changes that the intervention is hoping to achieve.	The causal links between outcomes in the pathway of change. It is the responses/cognitive shifts experienced by the beneficiaries of the intervention.	What you can measure to monitor progress and assess each step of the program's pathway to change.	Conditions beyond the control of the intervention that must be true for the outcome to be achieved. They are made explicit in a ToC.
Number of studies in which ToC component is included (%)	38 (100%)	38 (100%)	10* (26%) *A further 3 studies mention but do not describe	9* (24%) *A further 7 studies mention but do not describe	10 (26%)

## 9. Discussion

This scoping review provides a map of the literature for how ToC are used in CHSI. Many of the findings in this scoping review echo those found in Breuer, Lee, De Silva et al. (49) systematic review of public health interventions. Namely, that there is a wide variation in how ToCs are developed, utilised and refined, and the literature presents limited detail on the rationalisation and development processes. These variations may relate to the historical emergence of ToCs as a tool for theory-based evaluation within academia, but also used within business and management practice. When evaluators utilise a ToC they have to decide whether to align themselves more closely with the academic underpinnings of the tool, reading and referencing the works of Weiss, Rossi, Connell, Kubisch and Chen (7, 13, 50). The alternative is to freely adopt the elements of the tool that work for them, and use it more liberally for the purpose of their evaluation in a business consultant-like fashion, even if this sacrifices some of the original intended value of the ToC. This tension is potentially why we now see such disparate findings in how ToCs are used and why.

### 9.1. ToC components

**Figure 3:**
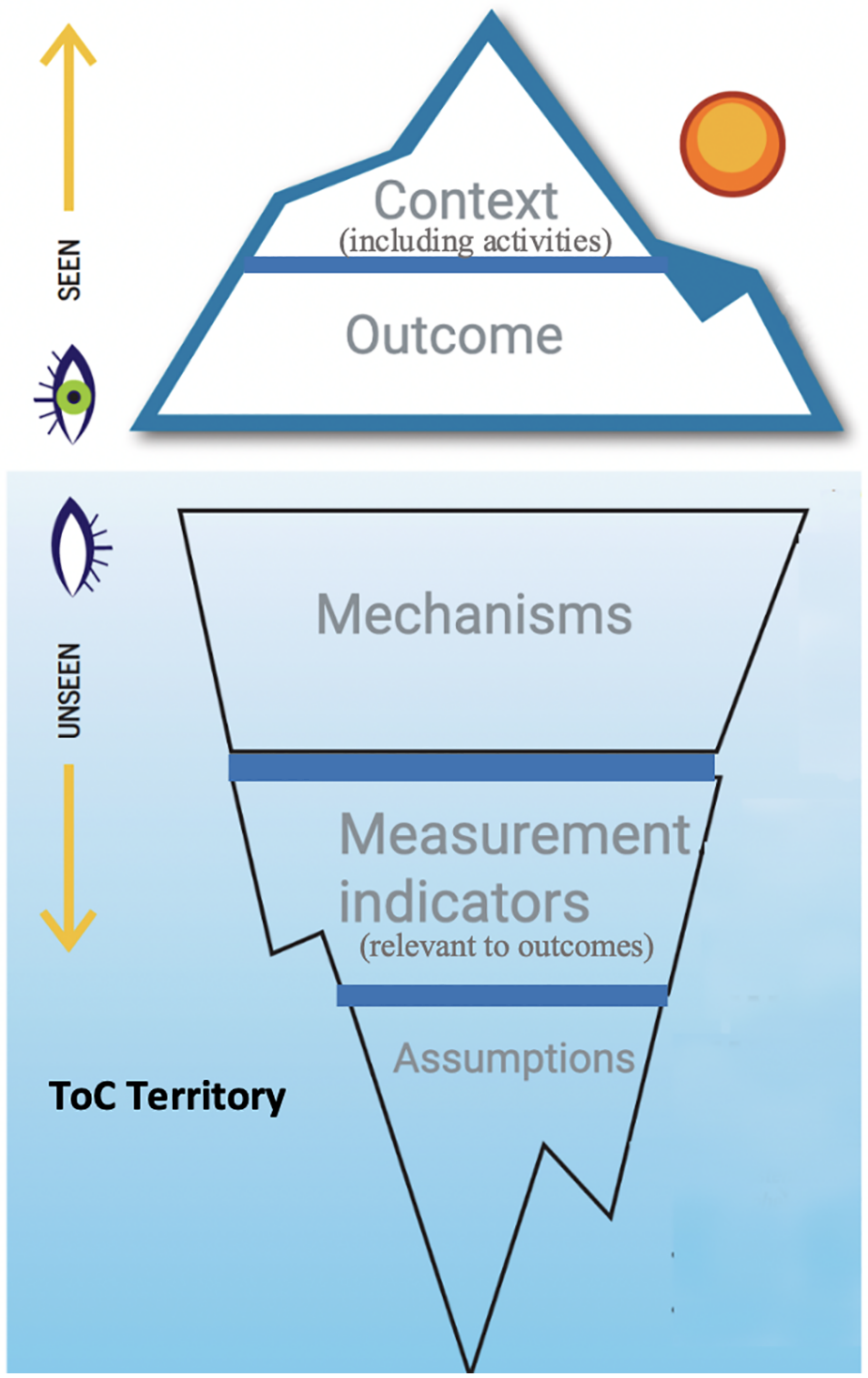
COMMA components of a ToC (adapted from Dhillon & Vaca 2018).

One aspect of ToC often discussed is what components are necessary to constitute a ToC. Currently, there is no clear and consistent classification of essential elements for a ToC, likely leading to the variation observed in the literature. As mentioned in the data extraction section of the methods in this paper, our classification of the components of a ToC, uses the acronym *COMMA*, and consists of the following elements; Context, Outcomes, Mechanisms, Measurement indicators, and Assumptions. Interestingly, in this scoping review, two of the five elements of *COMMA*, context and outcomes, were evident in every paper whereas the other three components were all present in less than a third. These other elements, namely mechanisms, measurement indicators, and assumptions, are arguably the elements that differentiate ToCs from logic models and add the most depth of thought and understanding to a ToC and hence their common absence could be considered a missed opportunity. In order to encourage integration of all three of these components into standard ToCs it may be worth adjusting Dhillon and Vaca (22)'s diagram in the simplified way seen in Figure 3.

### 9.2. ToC use over time

The findings of this review suggest that the number of child health organisations reporting a ToC is increasing (see Figure 2 above). In fact, all studies included in this review except one were published after Breuer, Lee, De Silva et al. (49) systematic review of public health interventions. This finding is consistent with the broader ToC literature which indicates that the use of a ToC is increasing (5, 51). Another, theory-based evaluation approach being used increasingly are realist evaluations. A realist evaluation seeks to answer the practical questions of *what works for whom, under what circumstances, and how* (52–54)? Realist evaluations are distinct from ToCs in several ways. For example, realist approaches have a specific explanation of causation that is informed by the paradigm of realism. The generative causation model explains that mechanisms are triggered only under specific contexts to produce outcomes (52–54). Whilst ToCs explore mechanisms they do not detail that these can only be triggered in certain contexts. The realist conceptualisation of causation leads to the analytical unit for realist evaluations: context-mechanism-outcome configurations which differs to the analystical unit in the ToC approach—the ToC diagram. Whilst out of scope of this particular review, exploring the synergies between ToC and realist evaluation approaches, building on the work done by Blamey and Mackenzie (16) will be important to add to our depth of understanding for both approaches and how they may complement one another. More broadly, with the increasing use of ToC and theory-based evaluations as a whole, there is an increasing need for a better understanding of this tool by child health practitioners, researchers, program implementers and policy makers.

### 9.3. ToC mechanisms or “program theory”

One of the areas that requires further understanding is the actual program theory. Weiss (13) defines program theory as “the *mechanisms* that intervene between the delivery of the program service and the occurrence of the outcomes of interest.” That is, what is the *true* cognitive shift or causal mechanisms that occur within the program participant. It is an element of theory-based evaluations that requires considerable reflection by program implementers. Weiss (13) and more recently, Rogers and Weiss (55) and Blamey and Mackenzie (16) found that whilst many organisations claim to consider program theory, in practice it is often not done, or, poorly done. This scoping review mirrored these findings with less than a third of ToCs including reporting on mechanisms. Thus, the authors of this paper propose that further consideration of how mechanisms are developed and by whom is needed.

### 9.4. Child health practitioners and ToC

A ToC can be a useful tool in the development and/or evaluation of CHSI. A ToC may aid in promoting an understanding of an intervention and enabling evaluative learning. In its true form, a ToC is not merely a diagrammatic summary of what was done or what is planned. More research needs to be done into which child health organisations benefit from a ToC and in what way. Another consideration that needs further exploration is how a ToC may contribute to organisational learning and, more broadly, learning health systems. Finally, the authors recommend the use of a checklist such as that presented in Breuer, Lee, De Silva et al. (49) to aid in the ToC process and provide some standardisation in this potentially disparate field.

### 9.5. Strengths and limitations

A strength of this review was the screening process. The screening process was broad at the title and abstract stage because the inconsistency in the use of the term ToC meant that only selecting studies based on if they used the term “Theroy of Change” would have resulted in missed studies. The findings and discussion of this review effectively highlight the perceived strengths as well as several broader concerns with ToCs. This review also builds on the work of Breuer, Lee, De Silva et al. (49) by: demonstrating that the inconsistent use of ToC terminology has continued since Breuer, Lee, De Silva et al. (49) publication in 2016 and extends from the public health interventions field into the child health service interventions field, demonstrating that a broader search strategy can be used and leads to the inclusion of relevant studies that do not specifically mention Theory of Change, and includes a more detailed discussion on causal mechanisms of change. Regarding limitations, since this review only included studies published in English, the scope is limited. During screening conducting and reporting an inter-rater reliability assessment prior to resolving the conflicts would have enhanced the validity of the study. The findings presented in this review are related to the child health context which was the scope of this review because of the area of interest and expertise of the authors. These findings may be true in other contexts, however this would require further research.

## 10. Conclusion

This scoping review offers a review of how ToCs have been used in CHSI and in doing so, reveals both the potential strengths and current weaknesses of the tool when used for this purpose. This has several potential implications. Importantly, for practitioners and organisations interested in CHSI, it maps how ToCs have been used to enact system changes in these settings, and provides suggestions as to how the use of ToCs might be improved upon. Additionally, for implementation researchers, it further elucidates how ToCs are used and described in the literature. This includes the perceived meaning and value of ToCs and what important components are typically missing in use to date. This, in turn, encourages consideration of how the definitions, processes and classifications of ToCs could be clarified. Ultimately, this scoping review demonstrates the ways in which practioners involved in CHSI can maximise the benefits of ToCs.

## Data Availability

The original contributions presented in the study are included in the article/**Supplementary Material**, further inquiries can be directed to the corresponding author/s.

## References

[1] Institute of Medicine Committee on Health Services Research. A working defintion of health services research. Washington, DC: National Academies Press (1994). Available at: https://www.ncbi.nlm.nih.gov/books/NBK231502/.

[2] Steinwachs DM Hughes RG. Advances in patient safety health services research: scope and significance. In: Hughes RG, editor. Patient Safety and Quality: An Evidence-Based Handbook for Nurses. Rockville (MD): Agency for Healthcare Research and Quality (US). (2008). 21328761

[3] LohrKSteinwachsD. Health services research: an evolving definition of the field. Health Serv Res. (2002) 37(1):7–9. 10.1111/1475-6773.0102011949927

[4] Barnhart DA Semrau KEA Zigler CM Molina RL Delaney MM Hirschhorn LR Optimizing the development and evaluation of complex interventions: lessons learned from the BetterBirth program and associated trial. Implement Sci Commun. (2020) 1:29. 10.1186/s43058-020-00014-8 32885188 PMC7427863

[5] Paina L Wilkinson A Tetui M Ekirapa-Kiracho E Barman D Ahmed T Using theories of change to inform implementation of health systems research and innovation: experiences of future health systems consortium partners in Bangladesh, India and Uganda. Health Res Policy Syst. (2017) 15(Suppl 2):109. 10.1186/s12961-017-0272-y 29297374 PMC5751673

[6] Center for Theory of Change. What is Theory of Change? (2021). Available at: https://www.theoryofchange.org/what-is-theory-of-change/

[7] Connell J Kubisch A. Applying a theory of change approach to the evaluation of comprehensive community initiatives: Progress. Prospects, and problems. New approaches to evaluat- ing community initiatives, vol. 2, theory, measurement, and analysis. Washington DC: Aspen Institute (1998).

[8] Weiss CH. Nothing as practical as good theory: exploring theory-based evaluation for comprehensive community-based initiatives for children and families. New approaches to evaluating community initiatives:, vol. 1, concepts, methods and contexts. Washington, DC: Aspen Institute (1995).

[9] De Silva MJ Breuer E Lee L Asher L Chowdhary N Lund C Theory of change: a theory-driven approach to enhance the medical research Council's framework for complex interventions. Trials. (2014) 15(1):267. 10.1186/1745-6215-15-267 24996765 PMC4227087

[10] Skivington K Matthews L Simpson SA Craig P Baird J Blazeby JM A new framework for developing and evaluating complex interventions: update of medical research council guidance. BMJ Br Med J. (2021) 374:n2061. 10.1136/bmj.n2061 34593508 PMC8482308

[11] Nilsen P. Making sense of implementation theories, models and frameworks. Implement Sci. (2015) 10:53. 10.1186/s13012-015-0242-0 25895742 PMC4406164

[12] Connell J Kubisch A Schorr L Weiss CH. New approaches to evaluating community initiatives: concepts, methods and contexts. New York: Aspen Institute (1995).

[13] Weiss CH. Theory-based evaluation: past, present, and future. New Dir Eval. (1997) 76:41–55. 10.1002/ev.1086

[14] Weiss CH. How can theory-based evaluation make greater headway? Evaluation Rev. (1997) 21(4):501–24. 10.1177/0193841X9702100405

[15] Anderson AA. The community Builder's approach to theory of change: a practical guide to theory development. New York: Aspen Institute Roundtable on Community Change. (2006).

[16] Blamey A Mackenzie M. Theories of change and realistic evaluation: peas in a pod or apples and oranges? Evaluation. (2007) 13(4):439–55. 10.1177/1356389007082129

[17] Rogers P Petrosino A Huebner T Hacsi T. Program theory evaluation: practice, promise, and problems. New Dir Eval. (2004) 2000:5–13.s 10.1002/ev.1177

[18] Jones B Nagraj S English M. Using theory of change in child health service interventions: a scoping review protocol. Wellcome Open Res. (2022) 7:30. 10.12688/wellcomeopenres.17553.1 35284641 PMC8881691

[19] Peters M Godfrey C McInerney P Soares C Khalil H Parker D. The Joanna Briggs Institute Reviewers' Manual 2015: Methodology for JBI Scoping Reviews. Adelaide, SA Australia: The Joanna Briggs Institute. (2015).

[20] Peters M Marnie C Tricco C Pollock D Munn Z Alexander L Updated methodological guidance for the conduct of scoping reviews. Jbi Evid Synth. (2020) 18(10):2119–26. 10.11124/JBIES-20-00167 33038124

[21] Ouzzani M Hammady H Fedorowicz Z Elmagarmid A. Rayyan-a web and mobile app for systematic reviews. Syst Rev. (2016) 5. 10.1186/s13643-016-0384-4 27919275 PMC5139140

[22] Dhillon L Vaca S. Refining theories of change. J MultiDiscip Eval. (2018) 14(30):64–87. https://assets.ctfassets.net/dsmojq7rshge/42gHh3lNgseO4kcEEamGqk/fdf20dd9db6f93b53875c0ce26a44751/Refining_Theories_of_Change.pdf

[23] Vogel I. Review of the use of ‘theory of change’ in international development. London, UK: Department for International Development (DFID) (2012).

[24] Cordova-Pozo K Hoopes AJ Cordova F Vega B Segura Z Hagens A. Applying the results based management framework to the CERCA multi-component project in adolescent sexual and reproductive health: a retrospective analysis. Reprod Health. (2018) 15:1. 10.1186/s12978-018-0461-3 29422099 PMC5806234

[25] Daruwalla N Jaswal S Fernandes P Pinto P Hate K Ambavkar G A theory of change for community interventions to prevent domestic violence against women and girls in Mumbai, India. Wellcome Open Res. (2019) 4:54. 10.12688/wellcomeopenres.15128.1 31489380 PMC6719749

[26] DiLiberto DD Staedke SG Nankya F Maiteki-Sebuguzi C Taaka L Nayiga S Behind the scenes of the PRIME intervention: designing a complex intervention to improve malaria care at public health centres in Uganda. Glob Health Action. (2015) 8:29067. 10.3402/gha.v8.29067 26498744 PMC4620687

[27] Gupta M Kaur M Chakrapani V Rana M Bosma H Van Schayck OCP Impact of a multi-strategy community intervention to reduce maternal and child health inequalities in India: a qualitative study in Haryana. PLoS One. (2017) 12(1):e0170175. 10.1371/journal.pone.0170175 28099465 PMC5242542

[28] Hanley T Sefi A Grauberg J Prescott J Etchebarne A. A theory of change for web-based therapy and support services for children and young people: collaborative qualitative exploration. JMIR Pediatr Parent. (2021) 4(1):e23193. 10.2196/23193 33749615 PMC8078682

[29] Kabongo EM Mukumbang FC Delobelle P Nicol E. Combining the theory of change and realist evaluation approaches to elicit an initial program theory of the MomConnect program in South Africa. BMC Med Res Methodol. (2020) 20(1):282. 10.1186/s12874-020-01164-y 33243136 PMC7691101

[30] Lalli M Ruysen H Blencowe H Yee K Clune K DeSilva M Saving lives at birth; development of a retrospective theory of change, impact framework and prioritised metrics. Globalization Health. (2018) 14:1-N.PAG. 10.1186/s12992-018-0327-z 29378667 PMC5789747

[31] MacKenzie M Shite J Berzins K Judge K. The Independent Evaluation of 'Starting Well': Final Report. In: Office CSs, editor. Edinburgh: The Scottish Executive, Chief Scientist's Office. (2005).

[32] Gadsby EW Hotham S Eida T Lawrence C Merritt R. Impact of a community-based pilot intervention to tackle childhood obesity: a 'whole-system approach' case study. BMC Public Health. (2020) 20(1):1–12. 10.1186/s12889-020-09694-2 33256660 PMC7708136

[33] Wilbur J Bright T Hameed S Kuper H Polack S Mahon T Developing behaviour change interventions for improving access to health and hygiene for people with disabilities: two case studies from Nepal and Malawi. Int J Environ Res Public Health. (2018) 15(12):2746. 10.3390/ijerph15122746 30563096 PMC6313611

[34] Zamboni K Singh S Tyagi M Hill Z Hanson C Schellenberg J. Effect of collaborative quality improvement on stillbirths, neonatal mortality and newborn care practices in hospitals of Telangana and Andhra Pradesh, India: evidence from a quasi-experimental mixed-methods study. Implement Sci. (2021) 16(1):1–17. 10.1186/s13012-020-01058-z 33413504 PMC7788546

[35] Fuhr DC Acarturk C Sijbrandij M Brown FL Jordans MJD Woodward A Planning the scale up of brief psychological interventions using theory of change. BMC Health Serv Res. (2020) 20(1):1–12. 10.1186/s12913-019-4778-6 PMC7449040 32847580

[36] Makowiecka K Marchant T Wuleta B Anuraag C Laboni J Liman A Characterising innovations in maternal and newborn health based on a common theory of change: lessons from developing and applying a characterisation framework in Nigeria, Ethiopia and India. BMJ Global Health. (2019) 4(4):e001405. 10.1136/bmjgh-2019-001405 31406587 PMC6666810

[37] Mbuthia F Reid M Fichardt A. Development and validation of a mobile health communication framework for postnatal care in rural Kenya. Int J Africa Nurs Sci. (2021) 14:100304. 10.1016/j.ijans.2021.100304

[38] Hurtubise K Brousselle A Camden C. Using collaborative logic analysis evaluation to test the program theory of an intensive interdisciplinary pain treatment for youth with pain-related disability. Paediatr Neonatal Pain. (2020) 2(4):113–30. 10.1002/pne2.12018 35548259 PMC8975192

[39] Hernandez M Hodges S. Applying a theory of change approach to interagency planning in child mental health. Am J Community Psychol. (2006) 38(3–4):165–73. 10.1007/s10464-006-9079-7 17001524

[40] Mulhern E Appleford G. Adolescents 360 evaluation process evaluation methodology. Adolescents. (2008) 360:1–22. https://www.itad.com/wp-content/uploads/2020/02/A360-Process-Evaluation-Protocol-Paper_Revised_August-2019_final-CLEAN-191028-1.pdf

[41] Nimpagaritse M Korachais C Meessen B. Effects in spite of tough constraints—a theory of change based investigation of contextual and implementation factors affecting the results of a performance based financing scheme extended to malnutrition in Burundi. PLoS One. (2020) 15(1):e0226376. 10.1371/journal.pone.0226376 31929554 PMC6957191

[42] van Belle SB Marchal B Dubourg D Kegels G. How to develop a theory-driven evaluation design—lessons learned from an adolescent sexual and reproductive health programme in West Africa. BMC Public Health. (2010) 10:741. 10.1186/1471-2458-10-741 21118510 PMC3001738

[43] Faiz T Saeed B Ali S Abbas Q Malik M. OR To ICU handoff: theory of change model for sustainable change in behavior. Asian Cardiovasc Thorac Ann. (2019) 27(6):452–8. 10.1177/0218492319850730 31189326

[44] Seward N Hanlon C Abdella A Abrahams Z Alem A Arayao R Health system StrEngThening in four sub-sSaharan African countries (ASSET) to achieve high-quality, evidence-informed surgical, maternal and newborn, and primary care: protocol for pre-implementation phase studies. Glob Health Action. (2022) 15(1):1987044. 10.1080/16549716.2021.1987044 35037844 PMC8765245

[45] Chandani Y Andersson S Heaton A Noel M Shieshia M Mwirotsi A Making products available among community health workers: evidence for improving community health supply chains from Ethiopia, Malawi, and Rwanda. J Glob Health. (2014) 4(2):020405. 10.7189/jogh.04.020405 25520795 PMC4267090

[46] Michaud-Letourneau I Gayard M Pelletier DL. Contribution of the alive & thrive-UNICEF advocacy efforts to improve infant and young child feeding policies in Southeast Asia. Maternal and Child Nutrition. (2019) 15:e12683. 10.1111/mcn.12683 30793546 PMC6519196

[47] Greenland K Chipungu J Chilekwa J Chilengi R Curtis V. Disentangling the effects of a multiple behaviour change intervention for diarrhoea control in Zambia: a theory-based process evaluation. Globalization Health. (2017) 13:1–18. 10.1186/s12992-017-0302-0 29041941 PMC5645837

[48] Milner KM Bernal Salazar R Bhopal S Brentani A Britto PR Dua T Contextual design choices and partnerships for scaling early child development programmes. Arch Dis Child. (2019) 104(Suppl 1):S3–S12. 10.1136/archdischild-2018-315433 30885961 PMC6557220

[49] Breuer E Lee L De Silva M Lund C. Using theory of change to design and evaluate public health interventions: a systematic review. Implement Sci. (2016) 11:63. 10.1186/s13012-016-0422-6 27153985 PMC4859947

[50] Chen HT Rossi PH. The theory-driven approach to validity. Eval Program Plann. (1987) 10(1):95–103. 10.1016/0149-7189(87)90025-5

[51] Mackenzie M Blamey A. The practice and the theory:lessons from the application of a theories of change approach. Evaluation. (2005) 11(2):151–68. 10.1177/1356389005055538

[52] Pawson R Tilley N. Realistic evaluation. London, UK: SAGE Publications (1997).

[53] Pawson R. The science of evaluation: a realist manifesto. London, UK: SAGE Publications (2013).

[54] Wong G Westhorp G Manzano A Greenhalgh J Jagosh J Greenhalgh T. RAMESES II reporting standards for realist evaluations. BMC Med. (2016) 14(1):96. 10.1186/s12916-016-0643-1 27342217 PMC4920991

[55] Rogers PJ Weiss CH. Theory-based evaluation: reflections ten years on: theory-based evaluation: past, present, and future. New Directions for Eval. (2007) 2007(114):63–81. 10.1002/ev.225

