# Supplementary material for: Improving child health service interventions through a Theory of Change: A scoping review
Source: Front Pediatr. 2023 Apr 6;11:1037890. doi: 10.3389/fped.2023.1037890 (PMC10115981; doi:10.3389/fped.2023.1037890)
Supplement: Supplementary file 2 [file Table2.docx]

### Search strategies

**Medline (Ovid)**

1 (infan* or newborn* or new-born* or perinat* or neonat* or baby* or babies or toddler* or minor* or boy* or girl* or kid or kids or child* or schoolchild* or adolescen* or juvenil* or youth* or teen* or pubescen* or pediatric* or paediatric* or peadiatric* or school* or prematur* or preterm* or "pre term*" or prepub* or pre-pub* or preschool* or pre-school* or kindergarten* or nursery* or preadolescen* or pre-adolescen*).tw. 3186401

2 adolescent/ or exp child/ or exp infant/ 3775679

3 (perinatal* or peri-natal* or antepartum or ante-partum or intrapartum or intra-partum or postnatal* or post-natal* or pregnan* or fetus* or foetus* or fetal* or foetal* or neo-nate* or preterm* or premature or pre-term* or premie* or preemie*).tw. 1023369

4 (stepchild* or step-child* or youngster* or PICU).tw. 8454

5 1 or 2 or 3 or 4 5531052

6 (theory adj2 change).tw. 1318

7 "theory of change".tw. 598

8 "change theory".tw. 618

9 ((theory adj2 change) or "program* theory" or "theory informed" or "intervention development" or "process evaluation*" or "theory-based evaluation*" or "outcomes hierarch*" or "theory-of-action" or "logic* model*" or "logic framework*").tw. 10661

10 6 or 7 or 8 or 9 10661

11 5 and 10 3499

12 limit 11 to english language 3446

**Global Health (Ovid)**

1 (infan* or newborn* or new-born* or perinat* or neonat* or baby* or babies or toddler* or minor* or boy* or girl* or kid or kids or child* or schoolchild* or adolescen* or juvenil* or youth* or teen* or pubescen* or pediatric* or paediatric* or peadiatric* or school* or prematur* or preterm* or "pre term*" or prepub* or pre-pub* or preschool* or pre-school* or kindergarten* or nursery* or preadolescen* or pre-adolescen*).tw. 678145

2 adolescent/ or exp child/ or exp infant/ 71811

3 (perinatal* or peri-natal* or antepartum or ante-partum or intrapartum or intra-partum or postnatal* or post-natal* or pregnan* or fetus* or foetus* or fetal* or foetal* or neo-nate* or preterm* or premature or pre-term* or premie* or preemie*).tw. 179106

4 (stepchild* or step-child* or youngster* or PICU).tw. 1743

5 1 or 2 or 3 or 4 742476

6 (theory adj2 change).tw. 381

7 "theory of change".tw. 213

8 "change theory".tw. 149

9 ((theory adj2 change) or "program* theory" or "theory informed" or "intervention development" or "process evaluation*" or "theory-based evaluation*" or "outcomes hierarch*" or "theory-of-action" or "logic* model*" or "logic framework*").tw. 3196

10 6 or 7 or 8 or 9 3196

11 5 and 10 1309

12 limit 11 to english language 1281

**EMBASE (Ovid)**

1 (infan* or newborn* or new-born* or perinat* or neonat* or baby* or babies or toddler* or minor* or boy* or girl* or kid or kids or child* or schoolchild* or adolescen* or juvenil* or youth* or teen* or pubescen* or pediatric* or paediatric* or peadiatric* or school* or prematur* or preterm* or "pre term*" or prepub* or pre-pub* or preschool* or pre-school* or kindergarten* or nursery* or preadolescen* or pre-adolescen*).tw. 3940430

2 adolescent/ or exp child/ or exp infant/ 3615397

3 (perinatal* or peri-natal* or antepartum or ante-partum or intrapartum or intra-partum or postnatal* or post-natal* or pregnan* or fetus* or foetus* or fetal* or foetal* or neo-nate* or preterm* or premature or pre-term* or premie* or preemie*).tw. 1267954

4 (stepchild* or step-child* or youngster* or PICU).tw. 16614

5 1 or 2 or 3 or 4 5792635

6 (theory adj2 change).tw. 1434

7 "theory of change".tw. 615

8 "change theory".tw. 704

9 ((theory adj2 change) or "program* theory" or "theory informed" or "intervention development" or "process evaluation*" or "theory-based evaluation*" or "outcomes hierarch*" or "theory-of-action" or "logic* model*" or "logic framework*").tw. 12108

10 6 or 7 or 8 or 9 12108

11 5 and 10 3753

12 limit 11 to english language 3684

**WHO Global Index Medicus**

(tw:(infan* or newborn* or new-born* or perinat* or neonat* or baby* or babies or toddler* or minor* or boy* or girl* or kid or kids or child* or schoolchild* or adolescen* or juvenil* or youth* or teen* or pubescen* or pediatric* or paediatric* or peadiatric* or school* or prematur* or preterm* or "pre term*" or prepub* or pre-pub* or preschool* or pre-school* or kindergarten* or nursery* or preadolescen* or pre-adolescen* or perinatal* or peri-natal* or antepartum or ante-partum or intrapartum or intra-partum or postnatal* or post-natal* or pregnan* or fetus* or foetus* or fetal* or foetal* or neo-nate* or preterm* or premature or pre-term* or premie* or preemie* or stepchild* or step-child* or youngster* or PICU)) AND (tw:("theory of change" OR "change theory" or "program* theory" or "theory informed" or "intervention development" or "process evaluation*" or "theory-based evaluation*" or "outcomes hierarch*" or "theory-of-action" or "logic* model*" or "logic framework*"))

**SCOPUS**

( TITLE-ABS-KEY ( infan*  OR  newborn*  OR  new-born*  OR  perinat*  OR  neonat*  OR  baby*  OR  babies  OR  toddler*  OR  minor*  OR  boy*  OR  girl*  OR  kid  OR  kids  OR  child*  OR  schoolchild*  OR  adolescen*  OR  juvenil*  OR  youth*  OR  teen*  OR  pubescen*  OR  pediatric*  OR  paediatric*  OR  peadiatric*  OR  school*  OR  prematur*  OR  preterm*  OR  "pre term*"  OR  prepub*  OR  pre-pub*  OR  preschool*  OR  pre school* OR  kindergarten*  OR  nursery*  OR  preadolescen*  OR  pre-adolescen*  OR  perinatal*  OR  peri-natal*  OR  antepartum  OR  ante-partum  OR  intrapartum  OR  intra partum  OR  postnatal*  OR  postnatal*  OR  pregnan*  OR  fetus*  OR  foetus*  OR  fetal*  OR  foet OR  neo-nate*  OR  preterm*  OR  premature  OR  pre-term*  OR  premie*  OR  preemie*  OR  stepchild*  OR  step-child*  OR  youngster*  OR  picu )  AND  TITLE-ABS-KEY ( ( theory  W/2  change )  OR  "program* theory"  OR  "theory informed"  OR  "intervention development"  OR  "process evaluation*"  OR  "theory-based evaluation*"  OR  "outcomes hierarch*"  OR  "theory-of-action"  OR  "logic* model*"  OR  "logic framework*" )  AND  TITLE-ABS-KEY ( "health service intervention*"  OR  "quality improvement project*"  OR  "health service program*"  OR  "staff*"  OR  "financ*"  OR  "implementation" )  AND NOT  TITLE-ABS-KEY ( "school" ) )

**CINAHL**

S1 (MH "Infant+")

S2 (MH "Child+")

S3 (MH "Adolescence+")

S4 TI infan* or newborn* or new-born* or perinat* or neonat* or baby* or babies or toddler* or minor* or boy* or girl* or kid or kids or child* or schoolchild* or adolescen* or juvenil* or youth* or teen* or pubescen* or pediatric* or paediatric* or peadiatric* or school* or prematur* or preterm* or "pre term*" or prepub* or pre-pub* or preschool* or pre-school* or kindergarten* or nursery* or preadolescen* or pre-adolescen* or perinatal* or peri-natal* or antepartum or ante-partum or intrapartum or intra-partum or postnatal* or post-natal* or pregnan* or fetus* or foetus* or fetal* or foetal* or neo-nate* or preterm* or premature or pre-term* or premie* or preemie* or stepchild* or step-child* or youngster* or PICU

S5 AB infan* or newborn* or new-born* or perinat* or neonat* or baby* or babies or toddler* or minor* or boy* or girl* or kid or kids or child* or schoolchild* or adolescen* or juvenil* or youth* or teen* or pubescen* or pediatric* or paediatric* or peadiatric* or school* or prematur* or preterm* or "pre term*" or prepub* or pre-pub* or preschool* or pre-school* or kindergarten* or nursery* or preadolescen* or pre-adolescen* or perinatal* or peri-natal* or antepartum or ante-partum or intrapartum or intra-partum or postnatal* or post-natal* or pregnan* or fetus* or foetus* or fetal* or foetal* or neo-nate* or preterm* or premature or pre-term* or premie* or preemie* or stepchild* or step-child* or youngster* or PICU

S6 TI ( (theory n2 change) or "program* theory" or "theory informed" or "intervention development" or "process evaluation*" or "theory-based evaluation*" or "outcomes hierarch*" or "theory-of-action" or "logic* model*" or "logic framework*" ) OR ( (theory n2 change) or "program* theory" or "theory informed" or "intervention development" or "process evaluation*" or "theory-based evaluation*" or "outcomes hierarch*" or "theory-of-action" or "logic* model*" or "logic framework*" )

S7 AB ( (theory n2 change) or "program* theory" or "theory informed" or "intervention development" or "process evaluation*" or "theory-based evaluation*" or "outcomes hierarch*" or "theory-of-action" or "logic* model*" or "logic framework*" ) OR ( (theory n2 change) or "program* theory" or "theory informed" or "intervention development" or "process evaluation*" or "theory-based evaluation*" or "outcomes hierarch*" or "theory-of-action" or "logic* model*" or "logic framework*" )

S8 S1 OR S2 OR S3 OR S4 OR S5

S9 S6 OR S7

S10 S8 AND S9

**Google search (grey literature)**

Child* "theory of change" filetype:pdf first 10 pages
