# Supplementary material for: Improving child health service interventions through a Theory of Change: A scoping review
Source: Front Pediatr. 2023 Apr 6;11:1037890. doi: 10.3389/fped.2023.1037890 (PMC10115981; doi:10.3389/fped.2023.1037890)
Supplement: Supplementary file 3 [file Table3.docx]

Scoping review title and abstract screening guide

| 1. Health service intervention? | 2. Target age 0-19? | 3. Theory of change? |
| --- | --- | --- |
| Is it a new intervention, or, changes to, an existing intervention e.g. funding, staffing, delivery mode? | Are more than half of the intervention’s target years between 0-19 e.g. 10-22? | Is there a mention of a ToC/logic model or synonymous term? OR Is there a mention of **how and why** or how an intervention SHOULD work? |
| Is the intervention being delivered by a health service e.g. hospital, community health worker or primary health centre? |  |  |
